# Farm Animal Serum Proteomics and Impact on Human Health

**DOI:** 10.3390/ijms150915396

**Published:** 2014-09-01

**Authors:** Francesco Di Girolamo, Alfonsina D’Amato, Isabella Lante, Fabrizio Signore, Marta Muraca, Lorenza Putignani

**Affiliations:** 1 Department of Laboratory Medicine, Bambino Gesù Children’s Hospital, IRCCS, Piazza Sant’Onofrio 4, Rome 00165, Italy; E-Mails: francesco.digirolamo@opbg.net (F.D.G.); marta.muraca@alice.it (M.M.); 2 Department of Chemistry, Materials and Chemical Engineering “Giulio Natta”, Politecnico di Milano, Via Mancinelli 7, Milano 20131, Italy; E-Mail: alfonsina.damato@polimi.it; 3 Department of Laboratory Medicine, San Camillo Hospital, Viale Vittorio Veneto 18, Treviso 31100, Italy; E-Mail: Isa.Lante@gmail.com; 4 Department of Obstetrics and Gynaecology, San Camillo Forlanini Hospital, Circonvallazione Gianicolense, 87, Rome 00151, Italy; E-Mails: anguela@libero.it; 5 Parasitology Unit, Bambino Gesù Children’s Hospital, IRCCS, Piazza Sant’Onofrio 4, Rome 00165, Italy; 6 Metagenomics Unit, Bambino Gesù Children’s Hospital, IRCCS, Piazza Sant’Onofrio 4, Rome 00165, Italy

**Keywords:** farm animal serum proteomics, animal welfare, human health

## Abstract

Due to the incompleteness of animal genome sequencing, the analysis and characterization of serum proteomes of most farm animals are still in their infancy, compared to the already well-documented human serum proteome. This review focuses on the implications of the farm animal serum proteomics in order to identify novel biomarkers for animal welfare, early diagnosis, prognosis and monitoring of infectious disease treatment, and develop new vaccines, aiming at determining the reciprocal benefits for humans and animals.

## 1. Introduction

Proteomics aims at identifying, quantifying, and characterizing the structure and functions of all proteins in a living organism, as well as assessing the modulation of these properties based on space, time, and physiological state.

**Figure 1:**
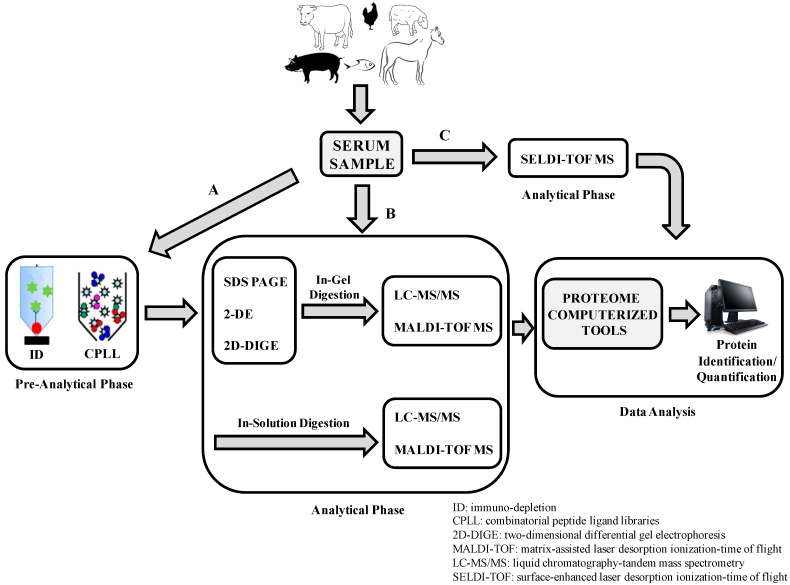
Routinely pre-analytical and analytical approaches for farm animal serum proteomic studies. Serum samples could be pre-treated (**A**) to reduce the high dynamic range in protein concentration, or not pre-treated (**B**,**C**) and, subsequently, analyzed through electrophoretic, chromatographic and mass spectrometric platforms. The large amount of data produced are, then, processed by sophisticated bioinformatic algorithms for qualitative and quantitative proteomic analysis.

Proteomic approaches on farm animal serum/plasma samples were applied with the purpose of certifying animal welfare in order to prevent a negative impact on human health (resulting in reciprocal benefits for humans and animals) and improve clinical management and treatment of human diseases [1] (Figure 1). Unfortunately, due to the lack of a complete characterization of animal genomes and the incomplete annotation of gene functions, studies on farm animal serum proteome are still in their infancy, compared to the fully mapped human serum proteome [2]. As shown in the next sections, to circumvent this problem, different strategies were applied by using homology searches or human annotations of BLAST-searched primary data.

Owing to the high dynamic range of serum samples (spanning ten orders of magnitude [3]) and the limited dynamic range of the analytical instrumentations (spanning five orders of magnitude [4]), the pre-analytical phase in animal serum proteomics field could play a crucial role in reducing the differences in serum protein concentration. Therefore, two serum pre-treatment methodologies have been applied for protein immuno-depletion (ID) [5], serum proteome equalization and low-abundance protein enrichment (combinatorial peptide ligand libraries technique (CPLL)) [6,7,8]. More than 90% of the serum proteome is represented by less than 10 different proteins (albumin, IgG, α1-antitrypsin, IgA, transferrin, *etc.*). Based on the high specific antibody, ID represents a very effective pre-analytical technique able to bind and remove any protein from the animal sera in a very effective manner.

The major criticisms of the ID technique are represented by the lack of suitable antibodies for all animal species and the accidental co-depletion of untargeted proteins [9]. Additionally, recent reports have strongly challenged such a protocol, stating that it might not enable access to the low-abundance proteome [10].

CPLL technique is able to significantly reduce the dynamic concentration range of the animal sera normalizing the concentration of the low-abundance proteins to those of the high abundance. Some weakness of the CPLL technique comprise the exclusion of the highly hydrophilic proteins from the capture event as well as the exclusion of other proteins designed as aberrant because they behave in an unpredictable way.

Both these techniques were successfully applied on the farm animal serum samples. The analytical phase includes proteomic approaches based on the electrophoretic, chromatographic and mass spectrometric technologies. In the field of animal serum proteomics both qualitative and quantitative methods have been applied, by using gel-based and gel-free mass spectrometry (MS) approaches.

The aim of qualitative proteomic analysis is to describe the proteins and their post translational modifications in serum samples, related to a specific patient status. The most widely used MS approach for in-depth serum protein identification is represented by the shotgun proteomics. It is a gel-free proteomic approach in which, after a total proteome enzymatic digestion, the produced peptides are separated with a high resolution liquid chromatography (LC) system and identified using tandem mass spectrometry (MS/MS) and automated database searching [11]. Qualitative analysis have also been performed by gel-based proteomic approaches, using one-dimensional (1-DE) and two-dimensional (2-DE) gel electrophoresis for protein purification and, LC–MS/MS or matrix assisted laser desorption ionization-time of flight-MS (MALDI-TOF-MS) [12] and automated database searching for a subsequent protein identification of the in-gel digested proteins.

Mass spectrometry based quantitative proteomics can be classified in relative and absolute measurements [13]. Aim of the relative quantification is establishing the modulations in protein expression between two (or more) serum proteomes (*i.e.*, healthy *versus* pathological subjects). 

Conversely, the absolute quantification aims at quantifying the amount of proteins/peptides into serum samples.

Animal serum proteomes have been analyzed only by using relative protein quantification approaches by: (i) gel-based platforms, 2-DE to compare spot patterns from different serum samples and two-dimensional differential gel electrophoresis (2D-DIGE) [14] to assess protein profile changes from two or three serum samples runs on a single 2D-gel; (ii) gel-free platforms represented by stable isotope based (Figure S1) and label-free methods. The first ones (ICAT [15] and iTRAQ [16], applied in the animal serum proteomic field, use stable isotopes to label digested proteins, introducing a mass variation between the labeled and unlabeled peptides in the process. The intensity signals obtained from the unlabeled and labeled peptide will then provide quantitative information in the MS spectrum.

Label-free methods [17] do not introduce labels and rely on chromatographic and MS data to quantify peptides in the samples. In this approach the separate LC–MS/MS runs of samples in different states are aligned and the quantitative analysis of protein concentrations is performed by calculating the area or the intensity of peaks of the same peptide in the two runs.

A very useful quantitative and qualitative MS method in the farm animal serum proteomic field is the surface-enhanced laser desorption/ionization time-of-flight (SELDI-TOF) mass spectrometry.

It combines two powerful techniques: chromatography to retain the proteins on a solid-phase chromatographic surface and MS to detect them by a TOF MS. SELDI-TOF is a profiling technique that, based on the relation between patient and control sera protein profiles, allows the identification of MS signals of potentially disease biomarkers. It represents a complementary visualization technique to 2-DE [18].

### Animal Genome Databases and Annotation of Gene Functions

As mentioned above, studies on animal serum samples have to deal with the partial characterization of animal genomes and the incomplete annotation of gene functions.

The main protein sequence and Gene Ontology (GO) Annotation consortium is Uniprot-GOA that is implicated in the cure of GO annotation program [19]. An application of GO is the creation of gene product annotations, based on literature evidences or sequence-based analysis. Actually, GO Consortium (GOC) has annotated about 100 million GO terms covering >400,000 species, including all the kingdoms of life. This number includes two classes of GO annotations: those created manually by reviewing the literature and those generated computationally via automated methods [20]. Uniprot provides the “complete proteome” for *Homo Sapiens* and “partially complete proteome” for *Gallus Gallus*, *Bos Taurus*, and *Sus Scrofa.* These well studied model organisms represent reference proteomes in several biomedical researches.

GOC aim is the development of a unique nomenclature, describing the functional characteristics (Molecular Function, Biological Process, Cellular Component) of any gene product from any organism [21].

The number of assigned GO terms to gene products and the number of distinct protein of human and main farm animals are showed in Table 1. The annotation data in GO database should contain information about the source (protein databases) and the evidence of implication in a specific biological process. The number of terms associated to a specific organism is then strictly related to the number of entries of gene products. Only few animal databases are quite complete and this is the main limit to describe the output of proteomic analyses of animal serum related to human serum.

However, even if there are some lacks in the full description of animal proteomes, their characterization is possible thanks to the use of reference proteome databases, homology searching (BLAST searching) and to interfere the reference GOA databases with experimental animal data. One of GO’s main uses is, in fact, to perform enrichment analysis on gene sets. For example, given a set of genes that are up-regulated under certain conditions, an enrichment analysis will find which GO terms are over-represented (or under-represented) using annotations for that gene set. There are a number of different tools that provide enrichment capabilities. Some of these are web-based, others may require the user to download an application or install a local environment. Tools differ in the algorithms they use, and the statistical tests they perform. Some other examples of enrichment tools are: gProfiler; BiNGO; Ontologizer and Cytoscape.

**Table 1:** Gene Ontology (GO) annotations of main farm animal species.

GO Annotation	Number of Associations	Number of Distinct Proteins
Human	403,735	46,159
Chicken	93,784	14,238
Cow	128,684	20,032
Pig	107,097	19,723

## 2. Animal Serum Proteomic Applications

As shown in Table 2, this article focuses on farm animal serum proteomics, aiming at determining the reciprocal benefits for humans and animals.

**Table 2:** Some applications of serum proteomic approaches in the veterinary field.

Farm Animals	Proteomic Techniques	Serum Proteomics Study	Reference
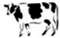 Bovine	2D-DIGE MALDI-TOF-MS MS/MS	Serum proteomic analysis of *Bruna* cows to identify novel potential biomarkers for stress and welfare	[22]
	MALDI-TOF-MS	Production of reproducible protein mass profiles able to identify calves undergoing illicit treatments	[23]
	2D-DIGE MALDI-TOF-MS	Investigation of new pathophysiological modulations in the plasma proteome of cows affected by milk fever	[24]
	2-DE MS	Study of serum proteome modulations of heifers during the last phase of pregnancy and early postpartum as a tool for the most accurate management of the peripartum period of these animals	[25]
	1-DE MALDI-TOF-MS	Identification of 480 bovine plasma proteins	[26]
	MALDI-TOF-MS 2-DE	Serum proteomics evaluation of cows for a better understanding of mastitis pathophysiology and an early diagnosis of the disease	[27,28]
	iTRAQ MS	Study of cattle serum samples infected with either *Mycobacterium bovis* or *Mycobacterium paratuberculosis* for discovery of potential biomarkers of infection and progression of disease	[29]
	CPLL 2D-DIGE LC–MS/MS	Early diagnosis of bovine Johne’s disease	[30]
	MALDI-TOF-MS 2-DE	Proteome and immunome of the tachyzoite stage of *Besnoitia besnoiti* in bovine besnoitiosis to study significant biological processes and host immune response associated with parasite infection	[31]
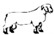 Ovine	2-DE MALDI-TOF-MS	Identification of novel staphylococcal antigens for rational vaccine design	[32]
	2-DE	Serological proteome analysis for the identification of *Staphylococcus aureus* antigens produced in the sheep immune response during mastitis	[33]
	SELDI-TOF-MS 1-DE LC–MS/MS	Identification of biomarkers in sheep paratuberculosis	[34]
	SELDI-TOF-MS	Detection of a panel of 4 putative serum protein biomarkers as immunological responses in infectious diseases in sheep	[35]
	2-DE MALDI-TOF-MS	Application of proteomics in putative biomarker discovery for early diagnosis as well as for monitoring the physiological and metabolic situations critical for ovine welfare	[36]
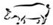 Swine	2D-DIGE MS	Evaluation of the validity of traditional stress biomarkers in pigs housed at different densities and identification of new potential stress biomarkers	[37]
	2-D-DIGE MALDI-TOF-MS LC–MS/MS	Identification of 10 differentially expressed protein spots by analysis of the serum proteome modulations in pigs infected by CSF virus *versus* uninfected controls	[38]
	LC–MS/MS	Identification of altered pigs serum proteome with FMD virus infection by analyzing the pigs before and after infection	[39]
	LC–MS/MS	Assessment of serum proteome profiles modulation between Ossabaw pigs and human NAFLD	[40,41]
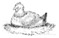 Poultry	2-DE MALDI-TOF-MS	Analysis of serum proteome of hens at different developmental stages as a base to explore the physiology of growth or reproduction of laying hens	[42]
	2-DE LC–MS/MS	Evaluation of APEC proteome changes after exposure to chicken serum to characterize specific protein molecules that may be involved in serum resistance of APEC isolates	[43]
	1-DE MALDI-TOF-MS	Evaluation of APEC growth in different host species requires different survival strategies	[44]
	2-DE, MALDI-TOF-MS	Evaluation of the serum proteome from broilers inoculated with one *Eimeria* species	[45]
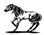 Equine	1-DE MALDI-TOF-MS MS/MS	Serum proteome evaluation of the effects of *Senecio jacobea* ingestion by horses	[46]
	MALDI-TOF-MS LC–MS/MS	Characterization of 29 different serum proteins to establish a horse serum protein database and acquire a better knowledge on equine proteome modulation during diseases	[47]
	SELDI-TOF-MS MALDI-TOF-MS	Evaluation of differences in serum proteome profiles of horses, donkeys and mules to determine possible metabolism-related differences	[48]
	2D-DIGE MALDI-TOF-MS	Discovery of 7 potential biomarker candidates to improve diagnosis and therapy of autoimmune uveitis	[49]
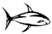 Fish	Capillary Electrophoresis–MS	Description of the *N*-glycans level in serum of salmon (*Salmo salar*) exposed to long-term handling stress	[50]
	2-DE LC–MS/MS	Assessment of the increase of lysozyme and angiotensin carboxypeptidase activities in plasma of fish transferred from hypoosmotic to hyperosmotic solution	[51,52]
	2-DE MALDI-TOF-MS	Determination of protein profiles of alterations in serum of rainbow trout (*Oncorhynchus mykiss*, *Walbaum*) as a measure of the acute phase response to the probiotics present in the feed	[53]
	2-DE MS/MS	Characterization of the alterations in serum acute phase response-related proteins with low molecular weight from loach after injury	[54]
	1-DE MALDI-TOF-MS	The first heterogeneous interactome between shrimp serum proteins and *Vibrio parahaemolyticus* outer membrane proteins	[55]

Abbreviations: MALDI-TOF-MS, matrix assisted laser desorption ionization-time of flight-mass spectrometric; 1-DE, one-dimensional electrophoresis; 2-DE, two-dimensional electrophoresis; 2D-DIGE, two-dimensional differential gel electrophoresis; SELDI-TOF-MS, surface-enhanced laser desorption/ionization time-of-flight mass spectrometry; LC–MS/MS, liquid chromatography–tandem mass spectrometry; CPLL, combinatorial peptide ligand libraries technique; CSF, classical swine fever; NAFLD, nonalcoholic fatty liver disease.

### 2.1. Monitoring of Animal Welfare

The expression “animal welfare” indicates their physical and psychological wellbeing. The assessment of animals’ wellbeing actually relies on behavioral tests (for most farm animals) which are easy to perform, or on measurement of stress markers like cortisol, also easy to measure but panels of biomarkers to associate each stress condition to physical and psychological state are desirable.

As described below, considerable experimental work, based on proteomics of farm animal serum, has been performed in order to assess the animals’ welfare by monitoring stressful states associated with their farming, food safety patho-physiological state and reproduction conditions. At present, these protein markers donot replace older routinely tests because they have yet to be validated.

Farms with insufficient space and difficult environments can stimulate a constant stress condition that alters the activity of the pituitary-adrenal axis with relapses on behavior, reproduction and food product quality [56,57,58].

To this purpose, different farm animal species represented by pig, cow and fish were studied. By using 2D-DIGE, immunoblotting, and MS technologies, Marco-Ramell *et al.* [37] assessed the validity of traditional stress biomarkers in pigs housed at different densities, identifying the actin as a novel potential stress biomarker and proposing cholesterol, pig-major acute phase protein, oxidative stress marker and cortisol as a molecular profile of the stress conditions in pigs.

Subsequently, with the same aim, the authors identified antioxidant enzymes, such as glutathione peroxidase, α2 Heremans Schmid glycoprotein, cholesterol, and fecal corticosterone, as novel potential biomarkers for monitoring the adaptation process of Bruna cows to difficult environments [22]. Moreover, Liu *et al.* [50], for the first time, described the *N*-glycans level in salmon serum (*Salmo salar*) exposed to long-term handling stress (15 s out of the water daily, for four weeks), compared with fish control sera. The results showed that the 83% of *N*-glycans in salmon serum is formed by mono-acetylated sialic acids, and the *O*-acetylation pattern of sialic acids was indeed altered by the handling stress. Finally, Jiang *et al.* [51] and Kumar *et al.* [52] revealed that plasma lysozyme and angiotensin carboxypeptidase activities were significantly enhanced in fish transferred from hypoosmotic to hyperosmotic solution.

Many other proteomic approaches were applied on cow serum samples to obtain results able to assess the bovine welfare. It is well known that performance enhancing agents are illegally used in animals, in order to increase lean meat production; however, these illicit substances could produce serious state of stress in the meat animals. Owing to the very low dosage and the use of uncharacterized new chemical agents, the identification of these molecules by conventional analytical approaches is challenging. This scenario encouraged a strong interest in the development of new and efficient molecular biomarkers for the detection of growth promoting agents in animals raised for meat production. In this regard, Della Donna *et al.* [23] developed a multivariate MALDI-TOF-MS proteomics platform, creating reproducible protein mass profiles from small volumes of serum samples, to identify calves undergoing illicit treatments. This pioneering work provided evidence of the potential application of MALDI-TOF-MS proteomic profiling in the food safety control. Xia *et al.* [24] investigated the plasma proteome of cows affected by milk fever (a disease of dairy cows characterized by reduced blood calcium levels, causing a state of stress and production of low quality milk) and identified the pathophysiological modulations of angiotensin, endopin 2B, albumin, fibrinogen β chain and IgG heavy-chain *C*-region by using DIGE technology, followed by MALDI-TOF proteins identifications.

Cairoli *et al.* [25] highlighted modulations in the serum proteome of heifers, to improve management of their peripartum period and, finally, for in-depth bovine plasma proteome characterization, Henning *et al.* [26] applied CPLL technology and three complementary fractionation strategies (SDS-PAGE, gel-free protein isoelectrofocusing or gel-free peptide isoelectrofocusing), identifying 480 bovine plasma proteins.

The application of proteomic technologies on horse serum samples produced different results in order to monitor equine welfare. Moore *et al.* [46] evaluated the effects of the ingestion of the *Senecio jacobea* (a poisonous weed ragwort very dangerous in hay) by horses, highlighting the presence of pyrrolizidine alkaloid toxins that are subsequently metabolized to cytotoxic pyrroles, which can bind proteins. They showed the modifications of equine plasma proteins with pyrrolic metabolites using sodium dodecyl sulfate polyacrylamide gel electrophoresis (SDS-PAGE), and MS as proteomics platforms. This model could be a new proteomic approach aimed at identifying potential protein biomarkers of ragwort exposure in horses and other livestock.

By establishing equine serum protein databases and by collecting knowledge on proteome modulation during diseases, Miller *et al.* [47] identified different serum proteins from different horse breeds by using 2-DE and MALDI-TOF-MS. Conversely, Henze *et al.* [48] evaluated the differences in serum proteome profiles of horses, donkeys, and mules, using SELDI-TOF technology and highlighted changes in posttranslational modifications (PTMs) of the protein transthyretin by using immunoprecipitation and MALDI-TOF-MS.

Fish serum proteomic studies also produced important results. Brunt *et al.* [53] applied proteomic platforms based on 2-DE and MALDI-TOF-MS to analyze the alterations of protein profiles in serum of rainbow trout (*Oncorhynchus mykiss*, *Walbaum*), as a measure of the acute phase response to probiotics *Aeromonas sobria GC2* and *Bacillus sp. JB-1*, present in the feed. Wu *et al.* (2004) [54] applied 2-DE with Tris–tricine gel system, peptide mass fingerprinting and MS/MS proteomic platforms to characterize the alterations in serum of low molecular weight acute phase proteins in loach after injury. In this experimental work, the authors highlighted the up-regulation of C-reactive protein and gastrin 71, the down-regulation of apolipoprotein and the disappearance of parvalbumin in serum from loach following injury with respect to control fish.

Finally, serum proteomic strategies have been applied by Huang *et al.* [42] in order to investigate the changes in proteome of laying hens at different developmental stages.

### 2.2. Monitoring of Diseases

Infectious diseases of animals are caused by pathogenic agents, such as bacteria, viruses, protists, and represent a major health threat.

Proteomic strategies have been applied on serum samples from bovine, ovine, swine, poultry, equine, and fish, to identify novel biomarkers for early diagnosis, prognosis, assessment and treatment monitoring of infectious diseases and to best understand their pathogenetic mechanisms and develop new treatments.

#### 2.2.1. Bovine Serum Proteomics

One of the first samples of applied infectious proteomic study is the analysis of bovine mastitis that causes decreased milk production and, therefore, economic damage to the industry. Bovine mastitis is an infectious disease, whose transmission is strongly influenced by factors related both to animals (breed, parity, stage of lactation, production level, morphological characteristics) and farming conditions (farm hygiene, presence of litter, conditions and maintenance of the milking machine, correct milking execution). Some bacteria involved in mastitis can cause serious human diseases. Moreover, treatment of the animal disease can result in the presence of antibiotic residues in milk, causing allergic sensitization or inducing antibiotic resistance in humans. The proteomic differential approach has been used in several works obtaining appreciable results [27]. In particular, the analysis of health bovine serum and mastitis serum by using 2-DE followed by MALDI-TOF-MS, allowed the authors to obtain disease-specific protein maps to monitor the course of the disease and the response to treatment [28].

Other bovine infectious diseases, that have been characterized by a proteomic approach, are tuberculosis, Johne’s disease (JD) and besnoitiosis. The bovine tuberculosis represents a highly prevalent zoonotic infection in cattle worldwide, caused by the intracellular bacterium *Mycobacterium bovis* (MB). Looking for an early detection of MB infection, Seth *et al.* [29] identified new protein biomarkers using iTRAQ on cattle serum samples infected with either MB or *Mycobacterium paratuberculosis* (MP).

JD is a worldwide common and economically relevant chronic inflammatory pathology of the small intestine of ruminants caused by *Mycobacterium avium* subspecies *paratuberculosis* (MAP). Moreover, numerous technological approaches currently available for JD diagnosis have a limited sensitivity. To this purpose, You *et al.* [30] applied CPLL, 2D-DIGE, and LC–MS/MS, to detect protein biomarkers of MAP infection in cows.

Finally, Bovine besnoitiosis (BB), a re-emergent disease in Europe, also present in Africa and Asia, is caused by the cyst-forming apicomplexan parasite *Besnoitia besnoiti* and is responsible for severe economic losses. However, the disease molecular mechanisms and parasite biology have still to be clarified. García-Lunar *et al.* [31] conducted a proteomic study to describe the proteome and immunome of the tachyzoite stage of BB using 2-DE, immunoblotting and MALDI-TOF/TOF MS.

They found several proteins, five of which were cross-reactive antigens between BB and *N*-*caninum*. They also investigated both the relevant biological processes and the host immune response associated with the parasite infection.

#### 2.2.2. Ovine Serum Proteomics

*Staphylococcus aureus* mastitis can also adversely affect the production of sheep’s milk.

Unfortunately, antibiotic treatment and prophylactic measures, including the development of an effective vaccine, so far have proven to be ineffective tools for the control of this disease. In this regard, Vytvytska *et al.* [32] and Le Maréchal *et al.* [33] applied 2-DE, immunoblotting and MALDI-TOF-MS proteomic approaches for the identification of novel staphylococcal antigens for rational vaccine design.

Zhong *et al.* [34] applied SELDI MS, SDS-PAGE, and LC–MS/MS technologies on sheep whole serum infected with MAP and identified transthyretin and α-haemoglobin as putative biomarkers.

Some papers have been published on the characterization of the early detection of the immunological responses in viral and bacterial infections in sheep using SELDI-TOF, 2-DE and MALDI-TOF/TOF MS techniques on sera. The results showed different up- and down-regulated proteins, related to controls that could be potential biomarkers [35,36].

#### 2.2.3. Swine Serum Proteomics

Sun *et al.* [38] analyzed the serum proteome modulations in pigs infected with classical swine fever virus, using 2-D DIGE followed by MALDI-TOF-MS or LC–MS/MS. They identified 10 differentially expressed protein spots, potentially biomarkers for the early diagnosis of the disease. Another serious infectious disease is foot-and-mouth disease (FMD) that affects cloven-hoofed animals. The virus causes high fever and produces blisters inside the mouth and on the feet that may break, thus causing lameness. FMD can affect humans but it is not dangerous for their health. Liu *et al.* [39] reported the first qualitative serum proteomic analysis from pigs before and after induced infection by FMD virus using LC–MS/MS. They developed novel drug targets and new biomarkers for care and early diagnosis of FMD and elucidated some mechanisms of the FMD virus pathogenesis.

Some human diseases can also be studied in animal models in order to compare the results with those from human studies and best understand the pathogenetic mechanisms and develop new treatments. The main limitation of this approach is related to the transferability of data between animal species. In fact, homologous proteins could have different post translational modifications and, therefore, different interaction protein patterns and biological functions, too.

Nonalcoholic fatty liver disease (NAFLD) is a common chronic disorder that occurs in the absence of significant alcohol abuse [59]; its pathogenesis has not yet been fully clarified. Appropriate animal models can help analyze the molecular mechanisms and identify new biomarkers for early detection and therapy monitoring. In this regard, Lee *et al.* [40] and Bell *et al.* [41] created a nutritional model of NASH (non-alcoholic steatohepatitis) and metabolic syndrome in miniature Ossabaw pigs very similar to human NASH and, using label-free quantitative proteomics approach, they assessed the modulation of serum proteomic profiles in animal and human NAFLD. The authors recommend the use of this pig model to improve knowledge on NASH and related metabolic abnormalities.

#### 2.2.4. Poultry Serum Proteomics

Each year the poultry industry suffers from consistent financial losses due to Colibacillosis disease, caused by an avian pathogenic *Escherichia coli* (APEC) that mainly affects broiler chickens resulting in septicemia and death. Using a proteomic approach, Tyler *et al.* [43] characterized specific proteins possibly involved in serum resistance of APEC isolates. In this study, the serum proteins were purified and separated by 2-DE approach; subsequently, ten protein spots, corresponding to eight differentially expressed proteins (some of them previously associated with *E. coli* virulence) were selected and characterized by LC–MS/MS. This pivotal study represented the precursor of future proteomic applications to identify the key players in serum resistance of APEC.

Later, Li *et al.* [44], using SDS-PAGE and MALDI-TOF/TOF-MS, compared the APEC proteome from human and avian sera, to determine growth in different host species and assess different survival strategies. In response to both human and avian sera, proteins contributing to active iron scavenging were up-regulated in APEC. Interestingly, these data support the concept that both human and avian sera are iron-limited environments.

Coccidiosis disease is characterized by destruction of the mucosa and is caused by *Eimeria* species, a protozoan responsible for a host- and infection site-specific intestinal disease. Two-DE, followed by MALDI-TOF/TOF MS were used to assess the serum proteome of broilers, after *Eimeria* acervulina inoculation. The identification of proteins that significantly changed in response to the infection allowed to highlight host response to coccidiosis and identify diagnostic and pharmacological targets [45].

#### 2.2.5. Equine Serum Proteomics

Proteomic strategies based on 2D-DIGE and MALDI-TOF approaches have been applied on healthy and uveitis-affected horse serum samples, with the aim of identifying seven biomarker candidates for the diagnosis of autoimmune uveitis [49].

#### 2.2.6. Fish Serum Proteomics

The mass spectrometry based proteomics have been applied by Liu *et al.* [55] to evaluate the first heterogeneous interactome between shrimp serum proteins and *Vibrio parahaemolyticus* outer membrane proteins; their results indicated that a complex network is formed when microbes invade the host.

## 3. Conclusions and Future Perspectives

Proteomics could help evaluate the farm animal welfare, reduce the risk of zoonoses and improve quality and safety of animal products while reducing financial losses.

During the last decade, the development of standard pre-analytical sample treatments, MS-based technologies and data processing systems for data management, have designated proteomics as a major platform to interrogate the plasma/serum proteome for discovery of next-generation biomarkers in the human and veterinary field. Due to the high number of serum proteins that may be simultaneously investigated, this approach is faster than ELISA and PCR techniques. The high initial investment required to buy the equipment represents a major limitation to a more widespread use of the technique. However, the costs involved are rapidly falling, thus, favoring its purchase. New proteomic technologies may dramatically improve discovery of next-generation biomarkers supporting diagnosis and monitoring of a variety of diseases in human and farm animals.

## Supplementary Materials

Supplementary Figure can be found at http://www.mdpi.com/1422-0067/15/9/15396/s1.

## Supplementary Files

Supplementary File 1
