# Supplementary material for: Farm Animal Serum Proteomics and Impact on Human Health
Source: Int J Mol Sci. 2014 Sep 1;15(9):15396–411. doi: 10.3390/ijms150915396 (PMC4200749; doi:10.3390/ijms150915396)

Supplementary Information

Figures S1. *Stable isotope labelling (SIL) as a powerful tool for quantitative farm animal serum proteome analysis*. ICAT (**Panel A**) technique labels the cysteines residues by a thio-reactive group and utilizes an isotopic linker region for heavy tag, and a biotin moiety for affinity based purification; Conversely, the isobaric tags for relative and absolute quantification (iTRAQ) (**Panel B**) label primary amines of either intact or proteolytically digested proteins. After labeling, samples are pooled and subsequently processed for MS analysis. Quantitation of SIL methods is based on the measures of LC–MS peak areas of the labeled peptides.

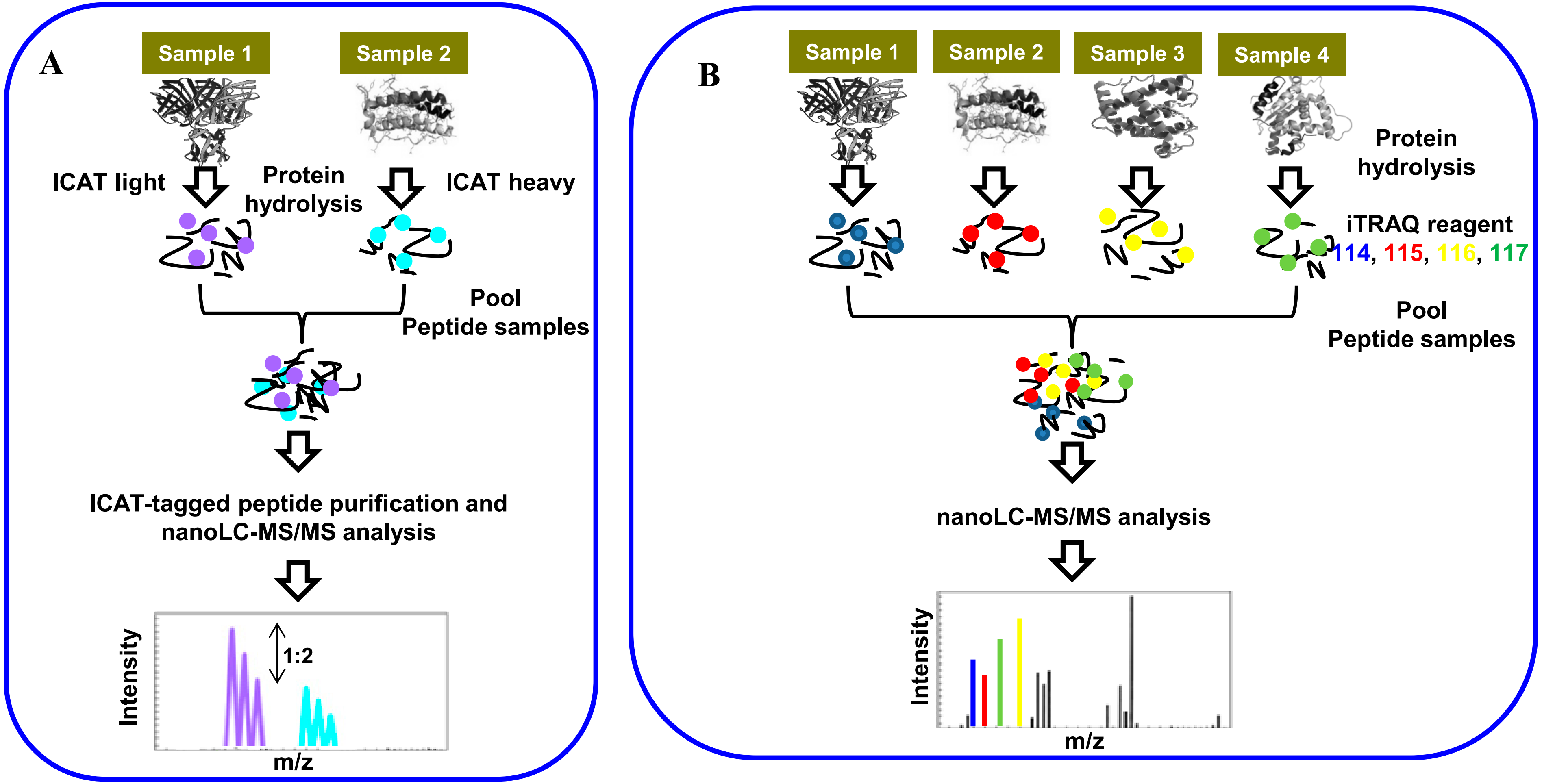

Supplement: Supplementary File 1 [file ijms-15-15396-s001.pdf]
